# Supporting a Family Member with Dementia to Live at Home: The Experiences of Caregivers

**DOI:** 10.3390/healthcare13020171

**Published:** 2025-01-16

**Authors:** Marcus Redley, Fiona Poland, Linda O’Raw, Martin Orrell

**Affiliations:** 1 School of Health Sciences, University of East Anglia, Norwich NR4 7TJ, UK; f.poland@uea.ac.uk; 2 Institute of Mental Health, University of Nottingham, Nottingham NG7 2RD, UK; linda.oraw@nottingham.ac.uk (L.O.); m.orrell@nottingham.ac.uk (M.O.)

**Keywords:** dementia, family carers, community care, carer experiences, qualitative interviews

## Abstract

Background: In the United Kingdom, specialist teams managing crises in dementia make efforts to reduce inpatient admissions by supporting people to live for longer in their own homes. However, fluctuations in the health and social circumstances of both the person with dementia and/or their family caregiver can lead to ‘crisis’: a breakdown in home care often leading to inpatient admissions. At this point, a team managing crises in dementia (TMCD) might become involved. These multidisciplinary teams begin with a rapid assessment to establish the needs of the person with dementia and their caregiver(s), followed by intensive but short-term interventions to reduce the risk of inpatient admission. There is little evidence as to how family caregiver experience input from a TMCD. Methods: A thematic analysis of qualitative interviews with caregivers supporting a family member with dementia to live at home and who have received support from a TMCD. Results: The personal troubles of family caregivers are intertwined with their experience of engaging with and trying to gain support from human services, particularly TMCDs. While respondents describe how specific individuals have addressed or added to the troubles they face, the most impactful interventions from their perspectives are medication for managing challenging behaviour and knowing that there is a service they can telephone in a crisis. Conclusions: Efforts to reduce inpatient admissions by supporting people to live in their own homes for as long as is practicable, even when carers may have reached the limits of their caring capacities, can, at best, only delay inpatient to long-stay residential accommodation. This is because when one frail older person has the responsibility of caring for another frail older person, even with support from TMCD and other health and social care services, crises are difficult to manage.

## 1. Introduction

In England, policies for the care and treatment of people living with dementia are geared toward supporting them to live in their own homes for as long as possible and reducing inpatient hospital admissions [1,2,3,4]. However, fluctuations in the health and social circumstances of both the person with dementia and their family caregiver can lead to a ‘crisis’: a breakdown in home care leading to inpatient admissions. In addition to significant costs, such admissions can result in the person concerned losing the ability to complete activities associated with daily living, such as dressing, getting into or out of a bed or chair, taking a bath or shower, or using the toilet [5,6,7], making the need for long-term residential accommodation more likely. It is not often the severity of a person’s dementia that predicts hospital admission, but other factors, which include multiple health conditions, polypharmacy, and level of dependency [8,9]. Consequently, efforts to reduce inpatient admissions have led to the widespread development of specialist clinical teams, which we refer to as teams managing crises in dementia (TMCDs).

These teams are multidisciplinary and might comprise psychiatrists, nurses, psychologists, occupational therapists, and healthcare assistants. They are usually provided by mental health services based in the community as either independent teams or as part of a community mental health team or memory assessment service. The typical mode of working for a TMCD is to begin with a rapid assessment to establish the needs of the person with dementia and their caregiver(s), followed by intensive but short-term interventions to reduce the risk of inpatient admission while long-term support is arranged with other community health and social care services. Although there is currently little research evidence and no guidance as to how a TMCD should be designed or operate [10], there is a substantial body of research on crisis where someone with dementia is living at home [11]. From a carer’s perspective, research suggests that caring for a person with dementia at home can be demanding and stressful, especially where dementia is accompanied by additional health problems, such as incontinence, physical dependence, or violent behaviour [12,13,14]. Although caregivers are often dedicated to supporting a spouse or family member at home, the efforts to do so can lead to self-neglect [6,12]. The most impactful interventions for carers are the medical management of challenging behaviour [13,15] and knowing that there is a service they can telephone in a crisis [16].

## 2. Method

The family caregivers in this qualitative study were purposively sampled to cover diverse carer relationships and recruited by practitioners working in the five NHS community mental health trusts that took part in the intervention arm of AQUEDUCT (Achieving QUality and Effectiveness in Dementia Care Using Crisis Teams). A randomised controlled trial (RCT) of a complex intervention [17] comparing usual care with the use of the AQUEDUCT Best Practice Resource Kit designed to support efforts to reduce both inpatient admissions and admissions to long-stay residential care [18]. Consent to be interviewed was sought by members of the participating TMCD, with the interviews, to optimise research costs and efforts, conducted over the telephone by MR. The interviews were semi-structured and based on illuminating three core areas: (i) the current situation after input from the TMCD, (ii) the circumstances that led to involvement from the TMCD, and (iii) the family caregivers’ expectations for the future. The planned conduct of these interviews aimed to take account of pressures on caregivers’ time, so they were kept brief, lasting on average 40 min. Where needed, unscripted follow-up questions were used to elicit additional details. These were also designed to cover the kinds of topics caregivers would be able to discuss comfortably in relatively short telephone conversations [19]. The interviews were transcribed verbatim (see Table 1).

The analysis of the interviews took participants’ views at face value (Silverman, 2001); no attempt was made to explore how respondents rhetorically constructed their answers [20] nor how those answers might have been influenced by the interaction between interviewer and interviewee [21]. Rather, the analysis was a two-stage process; participants’ answers to the three core questions were summarised question by question, allowing interpretative immersion in the data. These summaries were used as a basis for identifying new or emergent themes [22], which were developed, extended, and refined in analytic dialogue [23] between two authors (MR and FP). These emergent themes identified that (i) the experience of caregiving is embedded in human services, (ii) the main precipitators to TMCD involvement were behavioural, (iii) TMCD can both alleviate and aggravate the demands of caregiving, (iv) caregiving has its limits, and (v) the demands of being a caregiver do not end when a person moves to long-stay residential accommodation. The process of summarising and coding the data was supported by Microsoft Word (version 16.92), rather than specialist software designed to code qualitative data. We have followed the COREQ guidelines for reporting qualitative research [24]. Pseudonyms are used throughout.

## 3. Findings

The seven respondents’ (see Table 2) accounts were detailed accounts of ‘personal troubles’ [25] arising from the practical and emotional demands of caring. These accounts invariably included descriptions and their evaluations of a wide range of human services [26]: GP surgeries; NHS memory clinics; NHS in-patient care for physical and/or mental health conditions; adult social care, including providers of long-term residential care; and the NHS TMCDs, which were the specific focus of the AQUEDUCT trial. The people we interviewed did not make explicit that their personal troubles intersected with government policies to reduce inpatient admissions by supporting people to remain in their own homes for as long as practicable [1,2,3,4]. Yet, their accounts of caring for a family member provide many connections between their experiences as caregivers and the human services aiming to address the ‘social problem’ [25] of dementia by reducing inpatient admissions. These provide a resource that can contribute to the field by foregrounding carer designations of the nature, circumstances, and consequences of such connections for caregivers, the people being cared for, and the services involved in supporting them. The five emergent themes are discussed below.

### 3.1. Part One: The Experience of Caregiving Is Embedded in Human Services

Caregivers described their experiences of caregiving as not limited to their relationship with the person with dementia but also how their caregiving was bound up with services designed to support them. Describing how the TMCD first became involved, Mrs Darby recounts the circumstances of her husband’s dementia diagnosis and the telephone calls she made when he said he feared becoming violent towards her. When the interviewer assumes these telephone calls were made to her GP, Mrs Darby responds:

Excerpt 1:

Mrs Darby: You’re joking, our GPs don’t answer. No, what it was he’d been admitted to PR Hospital with a fever […] And then, so I’d been given some numbers […] what they called safety numbers, I think, that if there was a problem to ring up. […] So, all the numbers that I phoned in PR didn’t answer. So, in desperation I phoned up the Old People’s Mental Health and they said, “why ring us” and I said, “to be quite honest you’re the only person that’s answered the phone” and with that they sent somebody out and then of course they [the TMCD] got involved. (Mrs Darby caring for her husband)

In describing how the TMCD got involved and to answer the interviewer’s question, Mrs Darby reveals the distinctly under-responsive character of the services she has been involved with. Neither her GP’s surgery nor her hospital answered their telephones, despite the hospital having given her “safety numbers”. The only service to answer was the Older People’s Mental Health Team, and they questioned the legitimacy of her call, “why ring us”. To care for a person with dementia, in this case, a husband who fears he might become violent, is embedded with an experience of human services that either do not answer their telephone or question the legitimacy of that call. The experience of being a caregiver is also to recount and evaluate one’s experiences of human services (a theme explored further below). These respondents had a very particular or lay understanding [27] of the services they were involved with. To illustrate, Mr Darwin only knows the first name of the consultant, Elanor, who arranged for Jane, his long-term partner, to have a brain scan and refers to the medication she takes due to “getting very agitated and lashing out”, as “pills”. Moreover, after several attempts to ascertain which service has been supporting Mr Darwin, the interviewer observes, “it’s not necessarily that clear to you where the doctors, or the nurses, or the consultant, quite which service they’re in?” to which Mr Darwin replies:

Excerpt 2:

Mr Darwin: Well, yes, I suppose so. They’re all at a place called Hurricane House and that’s their base, and they’re sent out from there, the nurses, and obviously the consultant is there. When she needs to see—well, she came to the house a few months ago to see Jane, see how she is getting on with her new pills. The other girls, Sophie and Sharon, they’ve been coming round. They take blood and samples of wee wee and stuff. They’ve looked after her for quite a while. (Mr Darwin caring for long-term partner)

Mr Darwin cannot formally name the services he has been in contact with, nor which service was employing the consultant who has been seeing his long-term partner. Mr. Darwin provides a clear account of specific aspects of Jane’s care. He explicitly recalls the first names of individuals involved in supporting her, including Eleanor, identified as the consultant, and two women he refers to as “the girls”, Sophie and Sharon. Additionally, Mr. Darwin identifies the geographical location of their base, Hurricane House, and the interventions that have been undertaken. These interventions include conducting a scan, prescribing medication described as “pills”, and collecting various samples, including blood and urine, colloquially referred to as “wee wee and stuff”. This style of description, referring to practitioners by first names and using geographical references to identify services, was a routine feature of our data. Far from constituting a criticism of the respondents, this terminology signals how carers’ conceptualisations of services can differ markedly from the official names and identifiers used by these services. This also applies to their terms for medication, which they often referred to loosely as “pills”, or “tablets”. Notwithstanding the potential for confusion, our data enables the identification of specific troubles precipitating TMCD involvement.

### 3.2. Part 2: Behavioural Precipitators to TMCD Involvement

For all seven respondents, it was the behaviour of the person they were supporting, rather than physical demands or resource constraints, that was cited as leading to TMCD involvement. In the cases of Mr Hamilton and Mrs Darby, the TMCD became involved at the point where their spouses, after being detained in an Older Adults’ Mental Health Units, under the *Mental Health Act* 1983 [28], were about to be discharged. Detention under the *Health Act 1983* [28] is conditional upon an urgent need for treatment for a mental health disorder, where the person concerned is at risk of harming either themselves or others. For two other respondents, Mr Darwin (see Excerpt 2) and Mr Day, it was less clear which service precipitated TMCD involvement, but in both cases, these men were struggling to cope with challenging behaviours. Mr Darwin’s partner was “getting very agitated and lashing out”, while Mrs Day was acting aggressively towards her neighbours. In the case of Mrs Rose, it was her husband’s expression of suicidal thoughts that led to her seeking external help; the Older People’s Mental Health Team referred Mr Rose to TMCD. Mrs Thomas’ GP made a referral to the TMCD in response to Mr Thomas’s delusional belief that his clothes were wet, which was stopping him from getting dressed each morning. In the case of Mrs Shakespeare, the behaviour prompting TMCD involvement involved a complicated set of circumstances. Mrs Shakespeare lives alone and is supported by her two daughters, Fiona and Rachael Austin (the respondent). When Fiona was admitted to the hospital with pneumonia and Ms Austin was coming down with COVID, she arranged temporary accommodation for her mother in a local community residential home. As Ms Austin explains:

Excerpt 3:

Ms Austin: So, I get a phone call the next morning, and they [the residential home] went, “You’ve got to come and remove your mother. She’s been violent, she’s tried to smash the place up […] not good”. But I said, “I can’t come now I’m sorry”. So, my brother […] I had to phone him up and say, “Alex, you’ve got to fetch her, and you’ll have to look after her and bring her home”. Well, he did, and he said, “Well, I’m working. I can’t look after her. Can we put her in another home?” […]. So, they found a different home […] and then the same happened [Mrs Shakespeare behaved violently, and this residential home also refused to accommodate her]. So now, I don’t know what to do. I really didn’t know what to do. So, I phoned the Memory Clinic, I phoned the GP surgery. I just needed some help ‘til I was better. And oh, it took days. I phoned Social Services […] and eventually, I got a short-term intervention team [TMCD]. Somebody, god bless them, gave me their number. Oh, it was a nurse referred me at the hospital. (Ms Austin caring for her mother)

What is notable for carers is that TMCD involvement was precipitated by the challenging behaviours of the person being supported. Securing medication to manage these behaviours was seen by these respondents as overwhelmingly important.

### 3.3. Part 3: TMCD Can Alleviate but Also Aggravate the Demands of Caregiving

While caregivers identified how the TMCD had been able to appropriately support them, they also highlighted how TCMD input could also make demands on them. Praising the TMCD as “fabulous” Ms Austin (see Excerpt 3) reported that team members arranged to meet her at the bottom of her garden wearing masks (precautions against Covid). Here, it was agreed that team members would support Ms Austin’s mother for up to eight weeks with further support provided by a private care company until Ms Austin had recovered from Covid. Ms Austin described this arrangement as a “lifesaver”. Mrs Darby, Excerpt 4, described visits from the TMCD as “just talking basically and seeing how Derek was and asking him if he had any problems” This prompted the interviewer to ask, “did they do anything else other than sort of check up on you both and ask you how you were doing”:

Excerpt 4:

Mrs Darby: No, it was just talking.

Interviewer: Right. And was that something that you appreciated?

Mrs Darby: Yes, really it was, yes. It was knowing that you had support there and also, having their number that I knew I could phone. (Mrs Darby caring for her husband)

Carers said how much they appreciated this kind of support along with being provided with telephone numbers [14,29,30]. Despite the interviewer asking, none of the respondents reported that members of TMCD had suggested or been involved in changes to a person’s physical environment. Rather, the practical activities of the TMCD were specifically clinical: reviewing medications and, as reported in Excerpt 2, monitoring blood pressure and weight and taking samples of blood and urine. The data also revealed that contact with TMCD can on occasion add to, rather than alleviate, the concerns of family caregivers. Mrs Thomas elaborates on circumstances in which she felt unable to discuss with the TMCD her husband’s belief that his clothes were wet.

Excerpt 5:

Mrs Thomas: Well, the first time they came [members of the TMCD], two people came. So, it was possible […] for a very short period for me to speak to one of the team without him [her husband] being there. But after that, it wasn’t at all. Only ever one person came, and we all sat in the room together. So, the only thing I could do was sort of, if Harvey said something which actually wasn’t right, was just to shake my head briefly, or something like that. So, they never approached me to talk to me separately, so I wasn’t sure how much of the picture they were getting. (Mrs Thomas caring for her husband)

Mrs Thomas felt unable to publicly contradict her husband; consequently, she could not be sure the TMCD fully understood her predicament as someone caring for a person with delusional beliefs. While Mrs Darby, in Excerpt 6, describes, at the interviewer’s prompting, reasons why she had asked not to see a particular member of TMCD again:

Excerpt 6:

Mrs Darby: I thought she did the dirty work of a person called Liz Evan who is head of all of the departments apparently because Liz Evan was anti him coming home or anything, and when Liz Evans said to me if he comes home then he will have to be taken back into hospital he will have to go into a hospital at Trenchford which is miles and miles away. And then when she came, she said, “Well, you don’t see them every day anyway” and I said, “I might be strange, but I do see my husband everyday” so she said, “Well, we may be able to get a taxi occasionally for you”. And I said, “It’s not that, I could get a taxi, but I can’t do that everyday of my life” and it was just her attitude. (Mrs Darby, caring for her husband)

Our data contain several other examples where either the TMCD or other human services either aggravated the demands of caring or failed to meet expectations and so meant caregivers expended their limited time and energies without benefit to their caregiving situation. Examples included telephones not being answered, the withdrawal of the TMCD, which Mr Darwin described as being “let down”, or in the case of residential social care experience, the process of finding suitable accommodation as unpleasant because of the way this is performed, the available services being of poor quality, or when found, being too far away from the caregiver’s home to enable visits.

### 3.4. Part 4: Caregivers Have Limits

Closely linked to precipitating factors for TCMD involvement, carers all emphasised how they were confronted with what they experienced as limits to their capacity to care. Mr Darwin reveals that he is reaching his limits as a caregiver.

Excerpt 7:

Mr Darwin: Well I shall leave and find a flat or something. I don’t own the house, it’s Jane’s house […] I’ve said I’ll move out and find myself a flat […] whatever. And the Social Worker, I said […] “We’ll have to get a carer in then” I said “Yes, you’ll have to get a carer in and it will be a live in carer”. I don’t know if Jane will put up with that but that’s the situation is. They [adult social care] still won’t put her in a care home because she doesn’t want to go. (Mr Darwin caring for his long-term partner at home)

Mr Darwin’s options for managing or mitigating these limits are further constrained by the inability or resistance of Jane to alternative forms of care and support, such as live-in care or moving to a care home. Mr Hamilton’s wife is at home; however, at 89 years old, looking after her is bound to be physically demanding.

Excerpt 8:

Mr Hamilton: [the GP said] “Have you thought about putting her in a home?” and that’s a no, no, that’s where we are up to now anyway […] She [Mrs Hamilton] is stopping here as long as we can manage basically. And so, I can clean and wash, and I can do anything around the house. Although I’m knocking on in age but I’m pretty good for eighty-nine. (Mr Hamilton caring for his wife at home)

Mrs Darby describes the time when her husband was back at home before having to be re-admitted to the hospital:

Excerpt 9:

Mrs Darby: I managed the dementia reasonably well because we’d already been down this road with Terry’s mum […]. So, we’d already been down that road to a certain extent, but he became incontinent overnight when he left hospital […] and also, he became more disabled because they said [the TMCD] he’d lost the use of his legs at times, and they [the TMCD] said that that was the dementia, and also he’s got a heart condition that’s going downhill quite rapidly now. So, they [TMCD] said that was making him fall as well. So, it was like the two things that was making him fall, so that was the difficulty. And of course, I haven’t got the strength to lift him. So, that was a problem. (Mr Darby whose husband was waiting discharge to a residential care home)

Mrs Darby reported being less able to cope with her husband becoming incontinent and his falls. Mrs Darby signals her limits as a caregiver when she says, “I haven’t got the strength to lift him”. These excerpts show that caregivers have their limits and what these might be, but that when one frail elderly person supports another frail elderly person, the arrangement is likely to be especially precarious [29].

### 3.5. Part 5: Moving to Long-Stay Residential Accommodation

The demands on caregivers do not necessarily end when a spouse or partner moves into long-term residential accommodation. This was because they had to consider and anticipate factors when moving if they were to continue the caring relationship. While Mr Day says his wife is “very happy” in her current residential accommodation, he is, as he explains, trying to move her out of there:

Excerpt 10:

Mr Day: […] But I’m trying to get her out of there because it’s a good half-hour’s drive on an ordinary day to get there, and a week ago, Saturday, Sunday, when it was a very warm weekend, it took me over an hour and a half to get there, and to get back home, and hour and a quarter in the late afternoon. So, I’m trying to get her into another home in this area (Mr Day whose wife is living in a community residential home)

The time taken to visit a spouse living in residential accommodation is also a problem for Mrs Darby. She has already objected to one home, because as a non-driver, it would have taken her “five to six hours by bus” to get there, and in her opinion, “some of the homes are really dreadful”, and she will not have her husband “put anywhere”. Moreover, she describes the very process of identifying a community residential home for her husband was humiliating:

Excerpt 11:

Mrs Darby: […] unfortunately they [adult social services] put out a resume or something, whatever they call it, of Tony’s needs and different things and they said because of his needs and things nobody would take him. It’s really hard at the moment. I’m struggling at the moment with them because they make him sound like a monster, when he isn’t. (Mr Darby whose husband is waiting discharge to a residential care home)

Mrs Darby describes herself as “struggling at the moment”. No residential service is willing to take him, and it is difficult for her to accept how her husband is portrayed in the “resume” used to identify a suitable service: “they make him sound like a monster, when he isn’t”.

In summary, findings from our interview data reveal how the personal troubles of being a family caregiver for a person living with dementia are intertwined with their experience of engaging with and trying to gain support from human services, particularly TMCDs. These services seek to address the social problem of dementia by reducing inpatient admissions and supporting people to live in their own homes even when carers may have reached the limits of their caring capacities.

## 4. Discussion

This small sample of seven interviews can only provide an illustrative, rather than an exhaustive, account of the experiences of family caregiver receiving a service from a TMCD. Moreover, there is likely to be some selection bias in the sample, as recruitment was undertaken by members of TMCD. Nonetheless, this data provides a valuable resource detailing the personal troubles [25] of caregivers and their experiences of the human services, especially TMCD, tasked with reducing inpatient admissions by supporting people with dementia to live in their own homes for as long as is practicable.

Firstly, experience of caregiving was co-terminous with their experiences of human services. In one sense, it may not be so surprising, as this was a clinically framed sample, where the respondents had received a service from a TMCD. However, the interview data shows how and how much their experiences as caregivers are entwined with their experiences of many other services, GP practices (Excerpts 1 and 8), hospital inpatient care (Excerpts 1 and 9), or adult social care (Excerpts 3, 6, 7, 10, and 11). Carers seemed to have little idea whether they were receiving support from a specialist TMCD or one of several community services, as their understanding of services appears to be shaped by knowing a practitioner’s first name, the activities they undertake, and the name of the base from which they operate (Excerpt 2). As such, these respondents are perhaps poorly placed to explicitly evaluate specific services. Although not specific to dementia, this finding is consistent with the notion that older people often do not present themselves to services because many struggle to differentiate between services and therefore to distinguish which service might be appropriate to their needs [14]. What respondents can do is describe how specific individuals have addressed or added to the troubles they face. The most impactful intervention from carers’ perspectives was securing medication for managing challenging behaviour [13,15] and knowing that there was a service with relevant knowledge of living with dementia they could telephone in a crisis [16].

Although caregivers are often highly dedicated to supporting a spouse or family member at home, this responsibility becomes increasingly demanding and stressful when a person’s dementia symptoms are accompanied by additional challenges such as incontinence, physical dependence, or violent behaviour [12,13,14]. While the involvement of a TMCD can help maintain a person at home, it also places significant pressure on the carer and enables demands on them to persist even after the TMCD’s involvement ends. Hence, while TMCDs and other services do contribute to the goal of keeping people in their homes longer, the result is often that one frail older person is then left with the heavy responsibility of caring for another frail older person [31]. There is, perhaps, a tension between a caregiver’s understandable desire to care for their spouse (or partner) and their ability, in practice, to acknowledge the demands this entails. These demands and limiting caregiver opportunities to address them can result in self-neglect [6,12]. The number of frail older people living in the community, supported by a spouse or partner, who is also frail and elderly, continues to grow [31]. Caring at home under these conditions is therefore highly precarious [29] with a constant risk of breaking down, as there are clear limits to what caregivers can manage, contributing to carer fatigue and unmet needs [31].

Despite this, and in line with other studies, there is little evidence to suggest that enhancing the competencies of TMCDs will correspondingly reduce admissions to hospitals or residential care services [32]. Such insights highlight the importance of integrated work between services for older people’s mental health, primary care, social welfare, intermediate care, and hospital liaison [9].

While TMCDs and other services can help maintain individuals with dementia at home, caregiving remains highly burdensome, particularly when compounded by additional challenges such as incontinence or aggression. This often results in significant stress and self-neglect among frail, elderly caregivers, so creating precarious care arrangements. These findings suggest there is a disconnection between the skillsets of TMCD staff and patient needs, which can be attributed to the inherent limitations on how TMCDs operate. For example, TMCDs are not equipped to significantly improve the management of conditions such as incontinence, heart failure, or the frequent falls that are common among older populations. While TMCDs can prescribe medications for the management of challenging behaviours, which they routinely do once such conditions are identified, additional training for TMCD staff may be unlikely to lead to improved patient outcomes in areas that require the involvement of a broader array of services [13,31]. Hence, our data underscores the need for integrated, collaborative approaches across health and social care services to better support caregivers of people with dementia to address their unmet needs. Such broader, integrated services are above the capabilities of the TMCD to provide,

To fully recognise caregivers’ perspectives, it is crucial to take into account that the demands on them do not end when their family member or spouse moves out of the home into residential care. Neither are the demands on them confined within the home even when the person they care for is still living at home. Caregivers frequently experience the unsettling reality of watching their loved one’s identity and personality being defined by their health and behavioural conditions so as to make manageable their search for appropriate long-term care [33]. Even after securing a placement, caregivers often find themselves dedicating significant time and effort to travel for visits so as to maintain contact and safeguard the well-being of their loved one and may need to anticipate these when planning appropriate long-term care arrangements [12,14,34].

It is instructive to compare the situation in the UK to that of the Netherlands, where policies also promote reducing inpatient admissions by supporting people to live for as long as possible in their own homes. Practices for achieving the same ends in the two counties are, however, different. In the Netherlands, all persons living in the community will have a care manager whose role is to coproduce with the person with dementia and their family caregiver(s), the health and social care services needed for a person to remain in their own home. The expectation is that a care manager will be involved from diagnosis up until a person moves to a community residential service or dies. The care manager ensures that health and social care services respond to the changing needs of both the person living with dementia and their caregiver(s). Although this approach is costly [35] and not without problems where the interests of persons with dementia are in competition with and their caregivers [36], is distinctly different from the approach taken in the UK and seen in our data. In the UK, health and social care remain fragmented despite efforts at greater integration [37]. Furthermore, the provision of healthcare, while responsive to a crisis, is less sensitive to changes in the needs of persons with dementia and/or their caregivers. While the social care has shifted away from the provision of support, which increasingly falls on family caregivers, to the assessment of needs and service brokerage. In summary, we are still far from finding a sustainable model of service provision that enables persons with dementia to remain in their own homes for as long as is practicable and is also alert to the needs and wishes of caregivers.

## 5. Conclusions

The profound emotional strain of witnessing cognitive decline in the person they care for, coupled with their fear of becoming a burden to a life partner and how frequently they see the quality of costly residential care as questionable, all play a part in carers’ concerns about the sustainability of the care they can provide. These concerns arise, both within their caring relationships and also specifically when they have to navigate and negotiate within the complex context of local and national service pressures and provision. Whilst this sample was limited, the detailed range and nature of the experiences they articulate contributed grounded and contextualised details of these pressures from caregivers’ viewpoints. These can be used to contribute important insights to ongoing debates, notably those currently surrounding voluntary euthanasia and physician-assisted suicide [38]. Understanding the pressures on caregivers may help explain reasons for services’ capacity to respond to these, including TMCD, in being so constrained. These findings therefore have value for framing shared public and policy understandings of both the limits and the potential of current caregiving in the context of overlapping but resource-constrained services. The widely recognised reduction in NHS funding and social care over the past decade has eroded public confidence in the capacity of the system to provide adequate support. This decline can only exacerbate their concerns and uncertainties about the long-term reliability of the care systems upon which they depend. This study therefore provides insights into the pressures of caring and ways in which these can impact on caregivers’ own views of their capacities and choices to support a family member living with dementia.

## Figures and Tables

**Table 1:** Summary of features of interview study conduct and scope with family caregivers.

Type of Data	Semi-Structured Interviews with Family Carers; Audio Recorded and Transcribed Verbatim
Eligibility criteria	A family caregiver of a person living with dementia who had been seen by a TMCD in the past six months
Interviewer	Male postdoctoral researchers
Site and Duration of Interviews	Conducted over the telephone in the respondents’ own homes, average duration 40-min
Date of Data Collection	September to December 2023
Respondents	Seven Respondents, comprising, three wives caring for their husbands; two husbands caring for their wives; one male caring for his female partner; one daughter caring for her mother.
Interview Questions	(1) Current situation after input from a TMCD; (2) Circumstance that promoted involvement from a TMCD; (3) Family caregiver’s expectations for the future. Unscripted follow-up questions were used to elicit further details of respondents’ experiences.
Ethical Approval	Ethical approval was obtained from the NHS Health ResearchAuthority, ref: 16/WM/0273

**Table 2:** Summary of sample characteristics.

Name	Carer Caring for	Cared for Person’s Current Residence
Mrs Darby	Wife caring for husband	In general hospital awaiting discharge to community residential home
Mr Hamilton	Husband caring for wife	In own home
Mr Day	Husband caring for wife	Resident in nursing home considered too far away for regular visits
Mrs Rose	Wife caring for husband	In own home
Mrs Thomas	Wife caring for husband	In own home
Ms Austin	Daughter caring for mother	Mother living alone in own home
Mr Darwin	Male caring for long-term female partner	In own home

## Data Availability

The data presented in this study are available on request from the corresponding author due to ongoing data analysis.
